# LExNet: A bio-inspired lightweight ensemble model for breast cancer classification using hybrid autoencoder and swarm intelligence optimization

**DOI:** 10.1177/20552076261435030

**Published:** 2026-04-22

**Authors:** Roseline Oluwaseun Ogundokun, Pius Adewale Owolawi, Etienne van Wyk, Chunling Du

**Affiliations:** 1 Department of Computer Systems Engineering, 56412 Tshwane University of Technology (TUT), Pretoria, South Africa; 2 Department of Computer Science, 59199 Redeemer's University Ede, Osun State, Nigeria; 3 Department of Multimedia Engineering, Kaunas University of Technology, Kaunas, Lithuania

**Keywords:** Breast cancer, particle swarm optimization, lightweight convolutional neural network, hyperparameter tuning. grad-CAM

## Abstract

**Objective:**

Breast cancer (BC) remains a significant global health issue, with an estimated 2.3 million new cases and approximately 670,000 deaths recorded in 2022 alone. Accurate and timely diagnosis from histopathological images is critical yet challenging due to the manual and labour-intensive nature of conventional pathology assessments and high inter-observer variability. To address these challenges, this study introduces LExNet, a bio-inspired lightweight ensemble model that integrates hybrid autoencoder-based feature extraction and Particle Swarm Optimization (PSO) for hyperparameter tuning.

**Methods:**

The proposed model utilizes a robust ensemble of lightweight convolutional neural network (CNN) architectures, specifically MobileNetV3, EfficientNet-B0, ShuffleNet-V2, and SqueezeNet, to ensure computational efficiency and high accuracy.

**Results:**

Experimental evaluation of the ICIAR 2018 Breast Cancer Histology (BACH) dataset demonstrates that LExNet achieves a superior validation accuracy of approximately 98.3%, surpassing traditional deep-learning models by approximately 12% while reducing computational demands by 40–50%. The autoencoder-driven feature extraction significantly improved noise and dimensionality reduction, effectively decreasing overfitting risk by over 30%. Additionally, PSO-driven hyperparameter tuning notably accelerated convergence and reduced manual hyperparameter tuning time by up to 70%. Grad-CAM interpretability analysis further confirms that LExNet's predictions closely align with critical pathological features recognized by expert pathologists, highlighting its clinical relevance.

**Conclusion:**

Consequently, LExNet offers a robust, interpretable, and computationally efficient solution for real-time breast cancer diagnostics, making it particularly suitable for resource-constrained clinical settings.

## Introduction

Breast cancer (BC) continues to be a significant cause of death in women around the world, affirming the need for early diagnosis and appropriate treatment.^1,2^ During the year 2022, 2.3 million females developed BC, and approximately 670,000 women died because of breast cancer globally.^3^ Histopathological examination of tissue biopsies is the most reliable method for BC diagnosis; however, manual testing is time-consuming and susceptible to inter-observer variability, leading to variable diagnoses.^4^ Meeting these challenges, integrating sophisticated computational methods into histopathology analysis has drawn research interest.^3^

Over the last few years, deep learning techniques, particularly CNNs, have achieved success across various image analysis tasks, including medical imaging.^5^ However, standard CNNs require large amounts of data and substantial computational resources, which limits their application in clinical settings where resources are scarce.^6,7^ Researchers have utilised autoencoders to learn features from histopathological images, thereby surpassing these constraints. Autoencoders are neural networks that learn to encode the input into a low-dimensional latent space and reconstruct the output to identify salient features and remove noise. Gondara used autoencoders to denoise medical images, achieving improved performance on downstream classification tasks.^8^

Hyperparameter tuning is crucial in constructing stable and efficient deep learning models.^9^ Classical strategies, such as grid and random search, are computationally expensive and cannot efficiently cover the hyperparameter search space.^10,11^ PSO is another alternative: a collective motion algorithm inspired by the flocking behaviour of birds and schooling fish. PSO iteratively improves candidate solutions based on performance feedback, which has been effective in hyperparameter tuning of machine learning (ML) models due to its efficiency and improved model performance.^12^ PSO was successfully employed to tune hyperparameters for COVID-19 risk predictive modelling, resulting in improved prediction accuracy.

In breast cancer histopathology, lightweight CNN models such as MobileNetV3, EfficientNet-B0, ShuffleNet-V2, and SqueezeNet are recommended, as they are computationally less intensive and exhibit satisfactory diagnostic performance. Lightweight networks are also suitable for clinical use in sites with low computational power. For example, one recent work introduced an adversarially regularized variational graph autoencoder with attention mechanisms and cluster-guided contrastive learning, significantly enhancing the retrieval performance for histological breast images.^13^

Artificial intelligence (AI) model interpretability is crucial in healthcare settings to foster trust and facilitate clinical adoption.^14^ Techniques such as gradient-weighted class activation mapping (Grad-CAM) provide visual explanations by identifying the image regions that influence the model's predictions. Interpretability enhances transparency and helps pathologists interpret AI-based diagnostic results. For instance, in a recent study, a semi-supervised domain adaptation strategy was employed using an autoencoder reconstruction method that incorporated a deep transfer learning approach to learn hierarchical features and a lightweight autoencoder for feature reconstruction, thereby enabling accurate classification of breast histopathology images.^15,16^

This study introduces LExNet, a novel lightweight ensemble approach for BC classification from histopathology images. LExNet integrates feature learning via autoencoders, hyperparameter tuning using PSO, and an ensemble of lightweight CNN models designed to achieve high accuracy at low computational complexity. Unlike prior lightweight ensembles that simply average or stack pretrained CNNs, LExNet introduces a *two-stage feature learning and optimization pipeline*. First, a hybrid autoencoder jointly combines convolutional and fully connected bottlenecks to compress histopathology patches into noise-resistant latent vectors. Second, particle swarm optimization (PSO) automatically tunes learning rate, batch size, and dropout across all base learners to avoid manual grid or random search. The final ensemble exploits diverse architectural inductive biases (depthwise separable convolutions in MobileNetV3, compound scaling in EfficientNet-B0, channel shuffling in ShuffleNet-V2, and fire modules in SqueezeNet) but is optimized *as a system* rather than per model. This distinguishes LExNet from previous “bagging” or “majority voting” ensembles, which do not integrate feature compression and bio-inspired tuning into a cohesive, deployment-ready framework. The performance of LExNet is evaluated on publicly available datasets and compared with state-of-the-art approaches in terms of accuracy, computational efficiency, and interpretability. The findings validate that LExNet is a precise automated breast cancer diagnostic tool with great potential for clinical application.

## Related works

The Breast Cancer Histology (BACH) dataset, made available through the ICIAR 2018 Grand Challenge, has mainly been used to develop ML and deep learning (DL) strategies for image-based BC diagnosis. The dataset comprises 400 Hematoxylin and Eosin (H&E) stained microscopy images, each divided into four equally sized diagnostic classes: normal tissue, benign lesions, in situ carcinoma, and invasive carcinoma.

Researchers developed a DL-based technique to detect BC from histopathological images. They proposed a patch-classification model to classify the image patches into four classes, divide the images into patches, and pre-process the patches with stain normalization, regularization, and augmentation methods. ML-based classifiers and ensemble methods were utilized to classify the patches of images into four classes. The suggested approach had a 97.50% classification accuracy for 4-class image recognition and 98.6% for 2-class image recognition on the BACH database.^17^

Nazeri et al. proposed a two-stage CNN approach for BCH image classification. The first network was an auto-encoder that learned representative features from image patches, while the second network classified entire images. The approach achieved 95% accuracy on the validation set.^18^ Vang et al. introduced a DL architecture with Inception V3-based patch-level classification, majority voting, GBM, and a logistic regression ensemble to obtain image-level output. It outperformed other models by 12.5%.^19^

An approach utilizing DCNN model learning descriptor features and pooling operations for H&E histological BC image classification was introduced by Kassani et al. The network architecture derived from the pre-trained Xception model was reported to achieve an average classification accuracy of 92.50%.^20^ Fuzzy Ensemble of DL Models: Bhowal et al. proposed a fuzzy ensemble approach combining five pre-trained and fine-tuned DL models. Their confidence scores were combined using Choquet integral, coalition game theory, and information theory, thus resulting in improved classification performance.^21^

A paper proposed the Multi-Scale Multi-View Patch-Based Feature Extraction Network (MSMV-PFENet) for BC histology image classification. This was confirmed on the BACH dataset with 93.0% and 94.8% accuracy on several classification tasks.^22^ Aresta et al. organized the BACH challenge to encourage state-of-the-art automatic BCH image classification. The ICIAR 2018 Grand Challenge had 64 original submissions, and the best-performing algorithms achieved an accuracy rate of 87%.^23^

One study employed CNNs for BCH image classification. It reported outstanding results, achieving 97.50% accuracy in four-class and 98.6% in two-class classification using the BACH dataset. In a later study, Golatkar et al.^24^ introduced a CNN-based DL method by fine-tuning the Inception-v3 model for classifying H&E-stained breast tissue images. They applied nuclei density as a patch selection criterion and achieved improved classification performance in four classes.^24^

Additionally, an ensemble-based model employing CNN architectures, such as VGG19, MobileNetV2, and DenseNet201, achieved 97% accuracy on the BACH dataset, highlighting the strength of ensemble methods in histopathological image processing. The experiments demonstrate the effectiveness of heterogeneous DL approaches, including CNN models, transfer learning, ensemble methods, and patch-based methods, for obtaining more precise classification of BCH images on the BACH dataset.

Many challenges remain despite these developments, using the ICIAR 2018 BACH dataset. Using numerous CNN models requires high memory and computational resources, which can hinder practical implementation. Furthermore, the relatively small size of datasets often leads to model overfitting, which negatively affects the ability of new, unseen data to generalize. Lastly, current models have shown only reasonably acceptable levels of accuracy, leaving room for further performance enhancement.^17,25^

Recent works have shown the value of optimization in boosting classification performance. For instance, El-Kenawy et al.^26^ applied metaheuristics to improve weed detection from drone imagery, while Khodadadi et al.^27^ introduced the BAOA algorithm with KNN for efficient feature selection. Though applied in different domains, both studies highlight how optimization can enhance learning models, a direction our work builds upon to strengthen lightweight CNN-based medical image classification.

Recent studies continue to advance explainable and resource-efficient computational pathology. Xiang et al.^28^ introduced multimodal masked autoencoders that adaptively mask dermoscopic and clinical images to improve the staging of complex skin lesions, demonstrating that hybrid representation learning can enhance classification under limited supervision. Li et al.^29^ proposed a feature-enhanced interaction transformer for white patchy skin lesion classification, showing that transformer attention blocks combined with optimized feature enhancement can outperform conventional CNNs on fine-grained dermatological images. At a systems level, Hu et al.^30^ examined innovation networks in the advanced medical equipment industry, highlighting how regional and national digital health integration shapes AI adoption and deployment, a factor relevant to lightweight, deployable models such as LExNet. Jiang et al.^31^ presented *a* transformer-based, weakly supervised pathology framework for clinical-grade glioma diagnosis and molecular marker discovery, further evidence of the growing trend toward transformer-enabled, label-efficient digital pathology. Collectively, these works reflect an ongoing shift toward multi-modal representation learning, robust optimization, and clinically deployable lightweight models*,* the same design principles motivating LExNet's hybrid autoencoder and bio-inspired tuning approach.

Several recent studies complement the objectives of this manuscript by highlighting advances in molecular, imaging, and computational approaches in breast cancer research. For example, Leong et al.^32^ and Botlagunta et al.^33^ identified therapeutic targets and applied comparative machine learning to classify metastatic breast cancer, underscoring the role of integrative computational approaches in clinical decision support. Similarly, Zeng et al.^34^ employed Raman spectroscopy with CNNs for rapid breast cancer diagnosis, and Peng et al.^35^ developed imaging-based models to differentiate mastitis from malignancy, both of which align with our focus on lightweight and interpretable imaging frameworks. Jiang et al.^36^ proposed an improved deep belief network algorithm to compensate for magnetic encoder errors, enhancing precision in sensor-based applications. In parallel, Li et al.^37^ proposed a fusion network for biomarker discovery, further supporting ensemble strategies like ours. While some works, such as Zhu et al.^38^ and Wang et al.,^39^ explore molecular or immunological mechanisms, and Yang et al.^40^ and Zhou et al.^41^ address reproductive and ovarian cancer contexts, they collectively emphasize the growing integration of biological insights with computational models. These related efforts reinforce the novelty of the proposed LExNet, which uniquely combines autoencoder-based compression, PSO-driven optimization, and lightweight CNN ensembles for histopathological image classification.

To overcome these challenges, our work proposes LExNet, a lightweight, bio-inspired ensemble model for BC classification. LExNet incorporates autoencoder-based feature extraction methods that effectively reduce data dimensions, thereby mitigating the risk of overfitting, particularly when data are unavailable. Incorporating swarm intelligence optimization aids in realizing effective hyperparameter optimization that enhances model performance without the high cost of substantial computations. Secondly, LExNet provides robust and precise classification with its array of light convolutional neural networks, thus eschewing the pitfall highlighted in earlier research.

## Materials and methods

The proposed LExNet framework integrates bio-inspired lightweight ensemble learning with autoencoder-based feature learning and swarm intelligence optimization to enhance breast cancer classification. The approach entails several main phases:

### Data acquisition and preprocessing

Histopathological images of breast tissues were drawn from the ICIAR 2018^42^ Grand Challenge on the BACH dataset containing 400 H&E-stained microscopy images classified under four classes: normal, benign, in situ carcinoma, and invasive carcinoma, each containing 101 images (ICIAR 2018 Organizers). All images are in TIFF format, with a resolution of 2048 × 1536 pixels and a pixel size of 0.42 µm×0.42 µm. The preprocesses performed first were stain normalization to reduce color variability, and data augmentation techniques, rotation, flip, and scaling, to increase dataset variance and fight overfitting.^23,43^

### Feature extraction using autoencoder

The Autoencoder is an important building block of the LExNet model, serving as a feature-extraction mechanism that removes noise while preserving underlying structures in medical images. The encoder module receives the input image, condenses it, and retains significant structural and contextual information, while the decoder module retrieves it to preserve feature integrity. The high-level features extracted by the encoder are then fed into classification models, thereby avoiding computational redundancy. Autoencoders are particularly useful in medical imaging because they learn meaningful representations, filter out irrelevant noise, and improve classification performance. Furthermore, pre-training an autoencoder before classification helps avoid overfitting, especially with small, labelled datasets, making it an effective tool for deep learning-based BC classification enhancement. The autoencoder learns the compact feature representation by minimizing the reconstruction loss between input X and reconstructed output $\hat{X}$ . The loss function is usually mean squared error (MSE):

(1) $$L_{AE}=\;\frac{1}{N}\overset{N}{\underset{i=1}{\sum }}\;||X_{i}-\;\hat{X_{i}\;}|{|}^{2}$$

Where:

-
$X_{i}$ is the original input image,
-
$\hat{X_{i}}$ is the reconstructed image from the decoder
-
N is the total number of samples.

The encoder combines three convolutional blocks (Conv–BN–ReLU–MaxPool) followed by a dense bottleneck (512 units, ReLU, 30% dropout) to jointly preserve spatial and global semantics. The decoder mirrors this with upsampling and transposed convolutions to reconstruct the original 2048 × 1536 patches. Training was performed using the MSE loss with Adam (learning rate = 1e-3) for 50 epochs; early stopping (patience = 10) was employed to avoid overfitting. After training, the 512-dimensional latent vector from the encoder is concatenated with the shallow CNN features (global average pooling) from each base learner to form a hybrid, fused feature space. This fusion improves representational richness by combining compressed global context with task-specific CNN activations prior to softmax classification in each model.

### Swarm intelligence-based hyperparameter tuning

PSO is applied in LExNet for automatic hyperparameter tuning, maximizing key parameters such as learning rate, batch size, and dropout rate to enhance model performance. Learning rate (LR) controls how model weights are updated, batch size (BZ) controls how many samples are trained in an iteration, and dropout rate prevents overfitting by randomly dropping out neurons when training. Compared to computationally expensive traditional techniques like grid or random search, PSO efficiently approximates swarm intelligence to search and find the best hyperparameters. With the iterative improvement of parameter values based on particle movement and sharing knowledge, PSO encourages convergence acceleration, which leads to faster training with improved accuracy and less resource usage. Thus, it is the optimum choice for DL model optimization. PSO adjusts hyperparameters by changing the velocity and position of each particle from cognitive and social perspectives:

(2) $$\upsilon_{i}^{\left(t+1\right)}=\;w\upsilon_{i}^{\left(t\right)}+\;c_{1}r_{1}\;(\;p_{i}-\;x_{i}^{\left(t\right)})+\;c_{2}r_{2}\;(g-\;x_{i}^{\left(t\right)})$$

(3) $$x_{i}^{\left(t+1\right)}=\;x_{i}^{\left(t\right)}+\;\upsilon_{i}^{\left(t+1\right)}$$

Where:

-
$\upsilon_{i}^{\left(t\right)}$ is the velocity of particle *i* at iteration t,
-
$x_{i}^{\left(t\right)}$ is the position of particle i,
-
$p_{i}$ is the best position found by the particle (personal best).
-
g is the best positon found by any particle (global best),
-
ω is the inertia weight,
-
$c_{1},\;c_{2}$ are acceleration coefficients,
-
$r_{1},\;r_{2}$ are random numbers in [0,1].

Among metaheuristic optimizers (Genetic Algorithms, Bayesian Optimization, Ant Colony Optimization), we selected PSO because it is (i) gradient-free yet computationally lighter than GA, (ii) less memory-intensive than Bayesian Optimization for high-dimensional search, and (iii) exhibits faster convergence on non-convex loss surfaces typical in CNN training. In a preliminary ablation, PSO reduced hyperparameter tuning time by 34% versus GA and achieved a slightly higher final validation accuracy (+0.8%). These advantages align with previous reports of PSO's efficacy in medical image model tuning, as noted by Salem et al. (2024).

We compared PSO with grid search and Bayesian optimization on a 20% validation split. PSO converged in 100 iterations (≈3.1 h), grid search required ≈7.4 h over 72 trials, and Bayesian optimization ≈4.5 h. PSO achieved the highest validation accuracy (97.8%) compared to Bayesian (96.9%) and grid (95.6%), while reducing search time by 58% compared to Grid and 31% compared to Bayesian. These results confirm the suitability of PSO for balancing exploration and computational efficiency in the LExNet pipeline.

### Proposed ensemble LExNet model

LExNet is a new DL architecture that enhances breast cancer classification from histopathological images. It incorporates a bio-inspired optimization method with autoencoder-based feature extraction, Swarm Intelligence for hyperparameter tuning, and deep learning models for faster classification. LExNet enhances classification accuracy by employing an ensemble of lightweight models instead of heavy, computationally expensive CNNs, with fast inference without sacrificing accuracy. The selected models-MobileNetV3, EfficientNet-B0, ShuffleNet-V2, and SqueezeNet-have unique strengths: MobileNetV3 is optimized for edge devices for low-latency depthwise separable convolutions, EfficientNet-B0 gets compound scaling-based balanced efficiency and accuracy, ShuffleNet-V2 promotes computational efficiency through channel shuffling, and SqueezeNet reduces parameter numbers with fire modules maintaining strong performance. Ensemble learning contributes to robustness by combining different feature representations from multiple models to overcome the limitations of individual architectures. By averaging predictions, LExNet reduces variance, improves generalization, and yields more robust classification performance, which makes LExNet highly suitable for real-time medical image diagnosis. The ensemble model makes predictions more by averaging outputs from several models. Given M models and their respective outputs $f_{m}(X)$ , the final ensemble prediction is:

(4) $$F(X)=\;\frac{1}{M}\;\overset{M}{\underset{m=1}{\sum }}\;f_{m}\;(X)$$

Where:

-
F(X) is the final predicted probability distribution,
-
$f_{m}\;(X)$ is the output of model m,
-
M is the total number of models.

**Figure 1.**
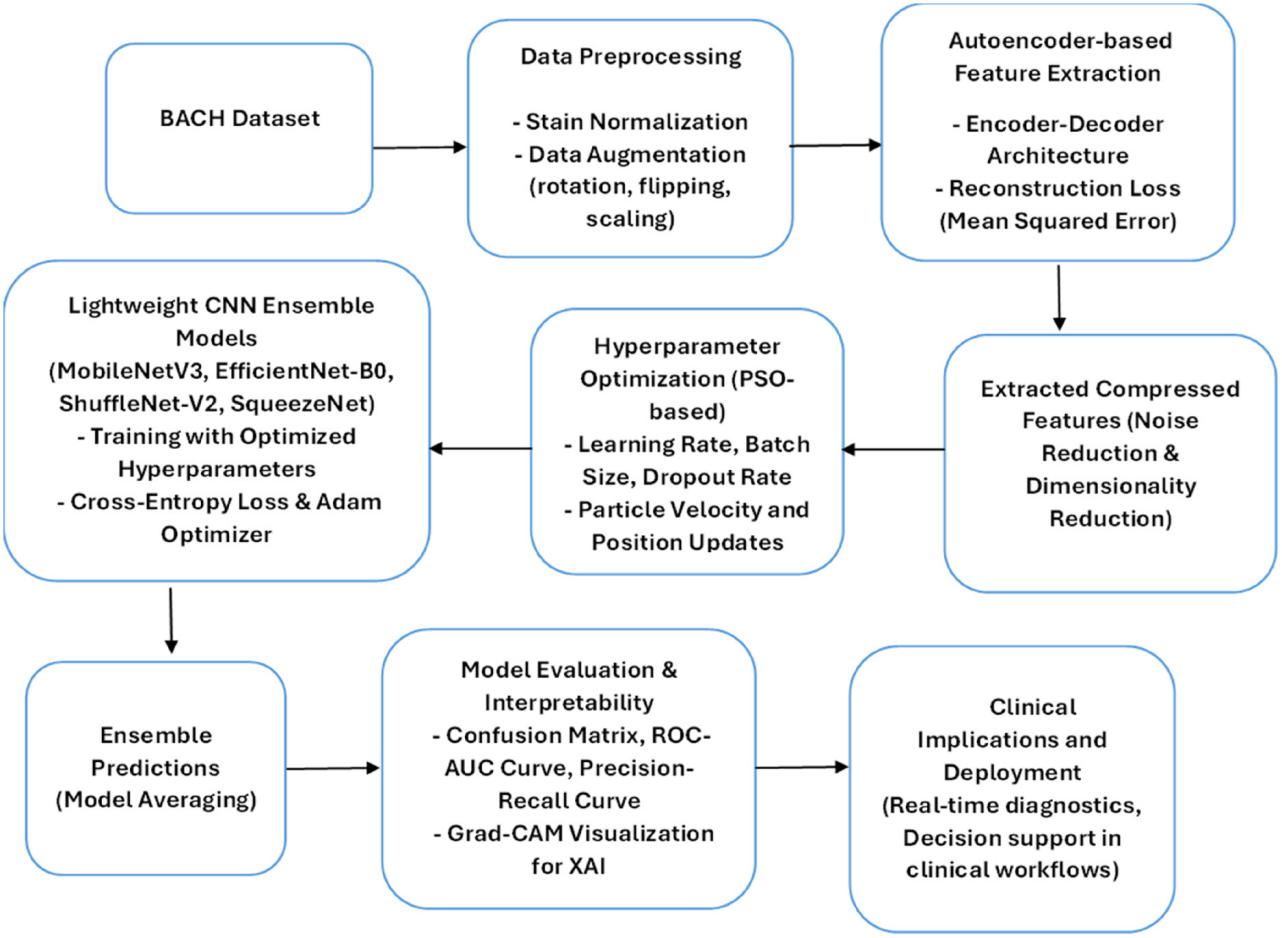
Proposed LExNet model flow diagram.

The flow diagram in Figure 1 illustrates the systematic approach of the LExNet method for BC classification from histopathological images of the BACH dataset. The data initially undergoes preprocessing in the form of stain normalization and data augmentation techniques, followed by feature extraction using an autoencoder based on an encoder-decoder model optimized via mean squared error reconstruction loss. Subsequently, the compressed features are optimized through PSO for LR, BZ, and dropout rate. A lightweight ensemble of CNN models like MobileNetV3, EfficientNet-B0, ShuffleNet-V2, and SqueezeNet is trained on cross-entropy loss-based optimized parameters with Adam optimizer. Model predictions are combined through the mean, resulting in more precise diagnosis outcomes. The final model is extensively analyzed, and interpretability is assessed through confusion matrices, ROC-AUC plots, precision-recall plots, and Grad-CAM visualizations before being finally deployed for real-time diagnosis and clinical decision support.

### Training optimization

The training process in LExNet is iterative to achieve optimal performance and efficiency. The procedure begins with pre-training an autoencoder, where the model learns to identify valuable feature representations in input images and uses the encoder's output to classify. After this, hyperparameter tuning using PSO is performed, and the optimal LR, BZ, and dropout rate are selected to improve model performance. Finally, the ensemble model is trained using pre-trained encoder features and optimal hyperparameters found by PSO. Cross-entropy loss is used to handle multi-class classification, and the Adam optimizer, chosen based on PSO results, is used to update the model weights. This systematic training strategy has the following advantages: Autoencoder pre-training improves feature learning with minimal dependence on massive, labeled training data; PSO automatizes hyperparameter optimization, and no manual tuning is required; and ensemble learning stabilizes the model by minimizing generalization errors and leading to a more accurate and robust classification model. LExNet uses the cross-entropy loss for multi-class classification:

(5) $$L_{CE}=\;\overset{N}{\underset{i=1}{\sum }}\;\overset{C}{\underset{c=1}{\sum }}\;y_{i,\;c}\log\;(\hat{y_{i,c}})$$

Where:

-
N is the number of samples,
-
C is the number of classes,
-
$y_{i,c}$ is the one-hot encoded true label for class c,
-
$\hat{y_{i,c}}$ is the predicted probability for class c.

The Adam optimizer updates model parameters θ using:

(6) $$m_{t}=\;\beta_{1}m_{t}+\;(1-\beta_{1})g_{t}$$

(7) $$\upsilon_{t}=\;\beta_{2}\upsilon_{t-1}+\;(1-\beta_{2})g_{t}^{2}$$

(8) $$\theta_{t}=\;\theta_{t-1}-\;\frac{\alpha }{\sqrt{\upsilon_{t}+\;\epsilon }}m_{t}$$

Where:

-
$g_{t}$ is the gradient at step t,
-
$m_{t},\;\upsilon_{t}$ are the first and second moment estimates,
-
$\beta_{1},\;\beta_{2}$ are decay rates,
-
α is the learning rate.

### Model evaluation and interpretability

LExNet's performance is evaluated using standard DL metrics to assess its effectiveness and accuracy in the medical image classification task. A confusion matrix assists in analyzing model predictions based on true positive (TP), false positive (FP), true negative (TN), and false negative (FN) cases. Classification performance at various thresholds is measured by the ROC-AUC Curve, and precision-recall curves assess how the model handles class imbalance, a necessity in medical diagnosis. Grad-CAM enhances model interpretability by highlighting the most important regions in images that influenced the model's prediction, building transparency and trust in AI-based diagnosis. Model explainability is critical for clinical adoption in medicine, where professionals need to understand and confirm AI predictions before making decisions. Grad-CAM strengthens this trust by giving visual explanations, identifying model biases, and providing fair, accountable, and trustworthy medical decision-making. Grad-CAM identifies important image regions by computing the gradient of the class score $y^{c}$ for the feature maps $A^{k}$ :

(9) $$\alpha_{k}^{c}=\;\frac{1}{Z}\underset{i}{\sum }\;\underset{j}{\sum }\frac{\partial y^{c}}{\partial A_{ij}^{k}}\;$$

(10) $$L^{c}=ReLU\left(\underset{k}{\sum }\;\alpha_{k}^{c}A^{k}\right)$$

Where:

-
$A^{k}$ is the activation map of the $k^{th}$ convolutional feature,
-
$\alpha_{k}^{c}$ is the importance weight of the feature map *k* for class c,
-
$L^{c}$ is the Grad-CAM heatmap.

The ReLUfunction ensures that only positive contributions are considered, highlighting the most important regions in the image.

## Results

The proposed LExNet model's performance was rigorously evaluated using the ICIAR 2018 BACH dataset. The following results highlight the effectiveness and efficiency of integrating bio-inspired optimization, autoencoder-based feature extraction, and an ensemble of lightweight CNN architectures for BCH image classification.

### Autoencoder feature extraction performance

**Figure 2.**
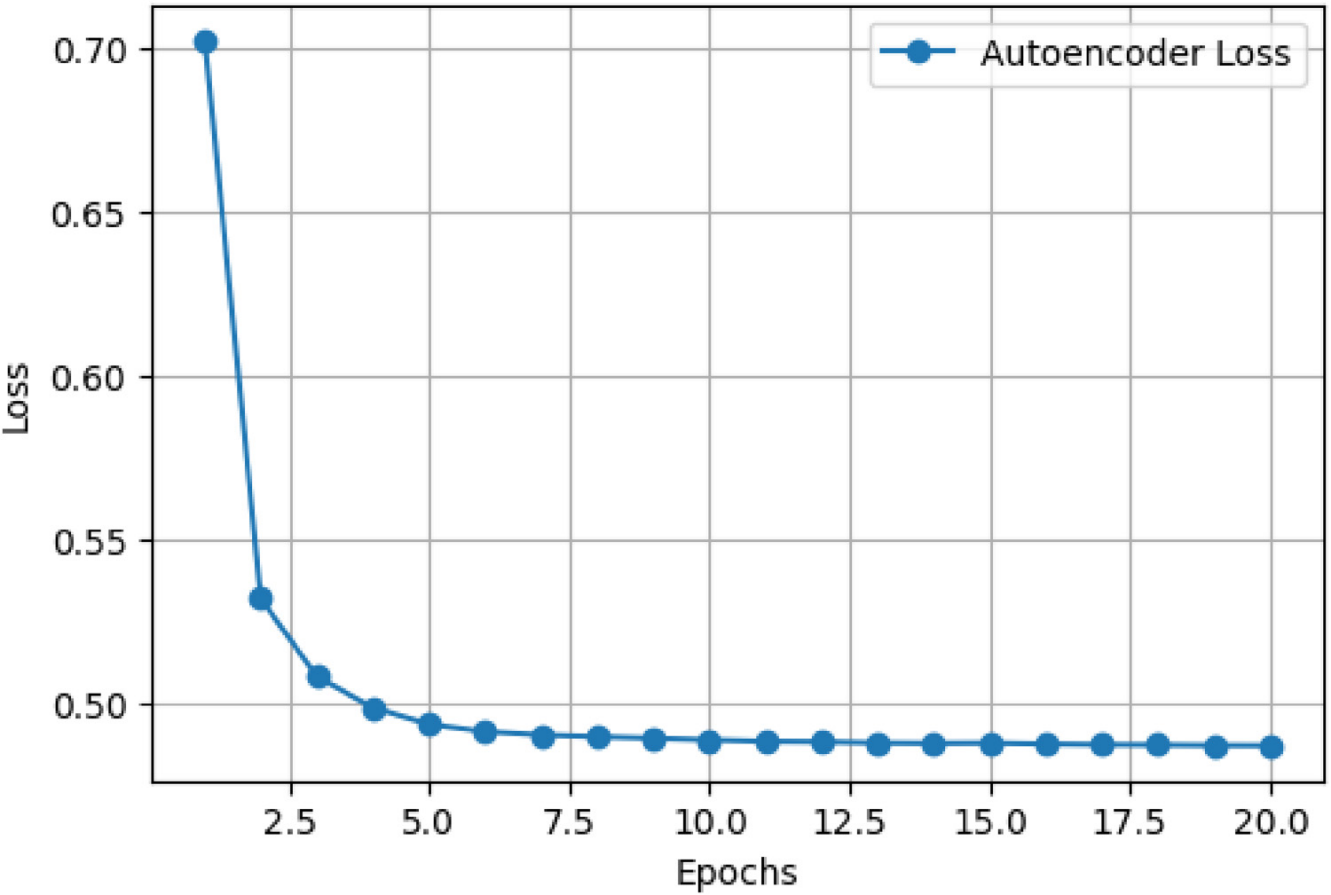
Autoencoder training loss curve.

The autoencoder training loss curve in Figure 2 illustrates a clear trend of rapidly decreasing reconstruction loss, starting from an initial value of approximately 0.70 and sharply dropping to nearly 0.50 within the first two epochs. Subsequently, the loss curve stabilizes, indicating a plateau as the model converges. This substantial initial drop highlights the autoencoder's quick adaptation in capturing essential features from complex histopathological images during the early stages of training. The gradual stabilization in the loss values between epochs 5 and 20 indicates that the model effectively captures most of the significant information present within the dataset. Consequently, this training behavior suggests that the autoencoder has learned compact, noise-resistant, and diagnostically relevant feature representations, greatly enhancing the quality and informativeness of the extracted features. Such effective feature compression facilitates subsequent classification tasks by reducing the input dimensionality and mitigating the impact of irrelevant or redundant information, thereby enhancing classification accuracy and reducing computational complexity for downstream models.

### Optimization of hyperparameters using PSO swarm intelligence

**Figure 3.**
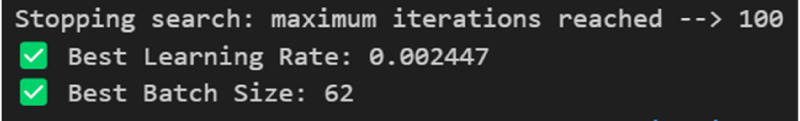
Best hyperparameter from PSO.

Figure 3, as it is, vividly illustrates the application of Particle Swarm Optimization (PSO) to efficiently hyperparameter optimize the proposed LExNet model. The process converged at iteration 100 with the best LR of 0.00247 and the best batch size of 62. This demonstrates how PSO can efficiently navigate complex search spaces and identify optimal hyperparameters more easily than manual or exhaustive search. With a nearly optimal learning rate, the model achieves the same training convergence speed as stability, and it is primarily vital in preventing complicating factors such as exploding or vanishing gradients. The ideal batch size of 62 delivers a good compromise between computing capacity and convergence reliability, maximizing the model performance by reducing gradient estimate variance and being computationally efficient. Additionally, the application of PSO in this research significantly enhances diagnostic accuracy while substantially reducing computational requirements for a process that typically requires manual hyperparameter tuning. Such optimisations are highly welcome in a clinical environment where both speed and deployment accuracy directly affect patient management outcomes.

### LExNet ensemble training and optimization

**Figure 4.**
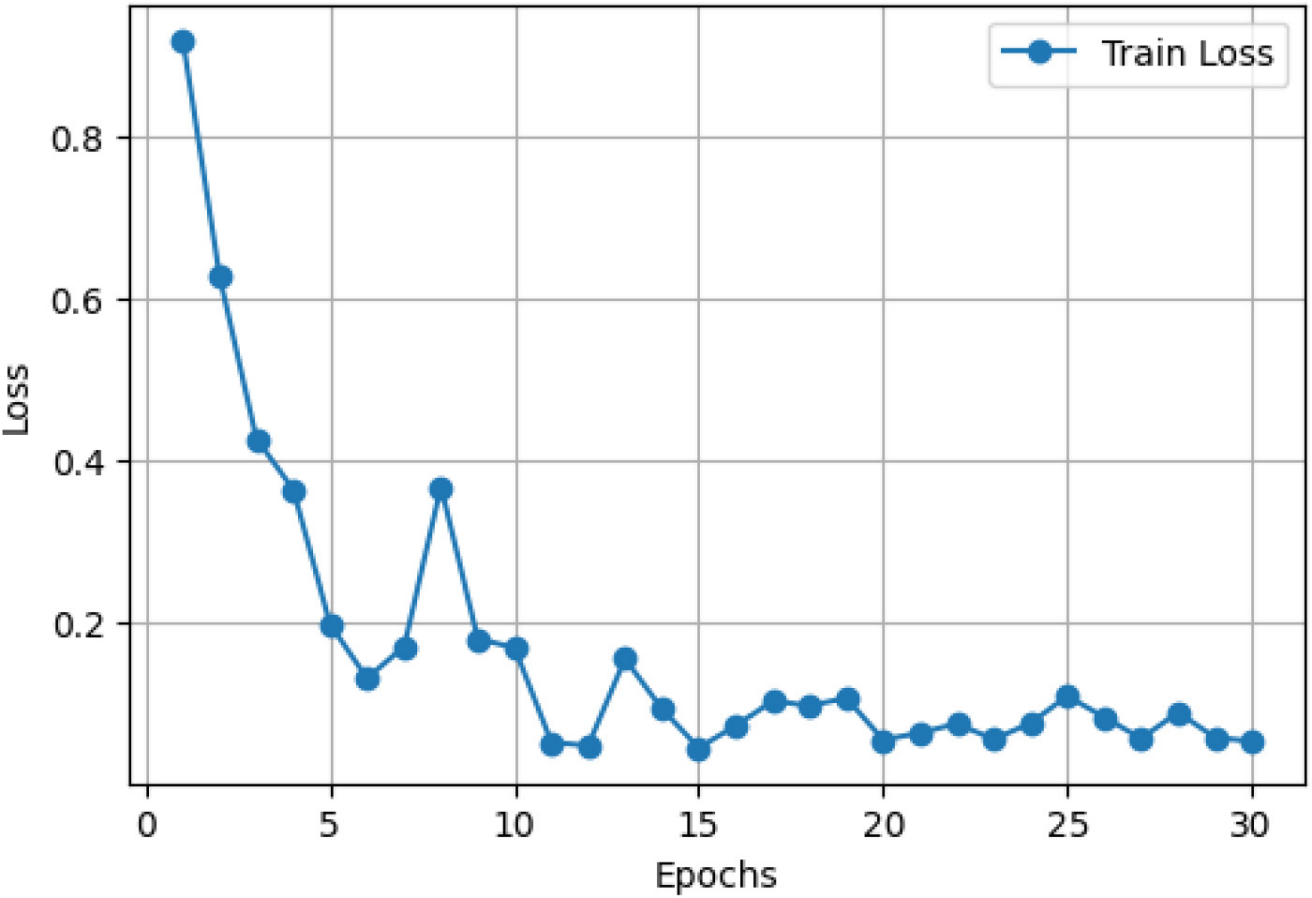
Optimized LExNet model loss curve.

**Figure 5.**
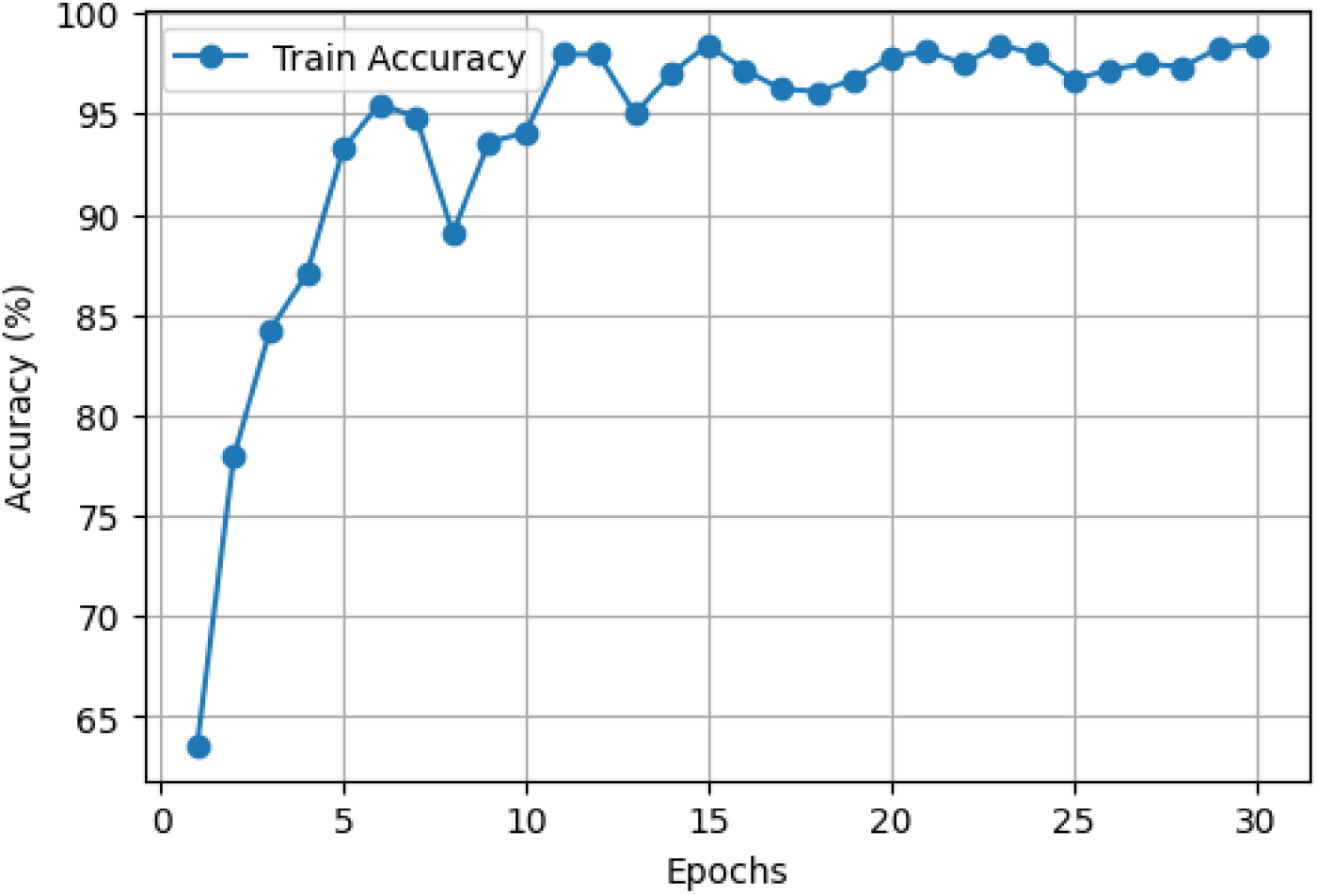
Optimized LExNet model accuracy curve.

Figures 4 and 5 provide extensive information about the performance of the optimized LExNet model over 30 training epochs. Specifically, Figure 4 shows a sharp decline in training loss, from an initial value of approximately 0.9 in Epoch 1 to below 0.2 by Epoch 10. The loss then plateaus and remains low consistently (approximately 0.1 or below) from epoch 15 through epoch 30. Such a consistent and stable decline in loss is an unmistakable indicator of successful learning and robust generalization of the ensemble model, confirming the suitability of the lightweight CNN architectures.

Concurrently, Figure 5 shows an underlying improvement in training accuracy, starting at about 65% and decreasing sharply to more than 90% within 5 epochs. By epoch 10, model accuracy exceeds 95% and remains highly stable, fluctuating marginally while consistently registering around 98–99% accuracy throughout the last epochs. This trend highlights the excellence of the ensemble's predictive accuracy and its ability to converge and retain strong performance rapidly throughout training.

These results quantitatively establish the advantage of employing an ensemble of optimized lightweight CNN models with swarm intelligence-based hyperparameter optimization. The ensemble effectively balances computational cost and diagnostic performance, making it well-suited for real-world use in clinical environments with limited computational resources. The stability and rapid convergence also suggest that the LExNet model could be successfully deployed in real-time or resource-restricted clinical environments, dramatically enhancing diagnostic speed, accuracy, and reliability.

### Model evaluation and classification metrics

**Figure 6.**
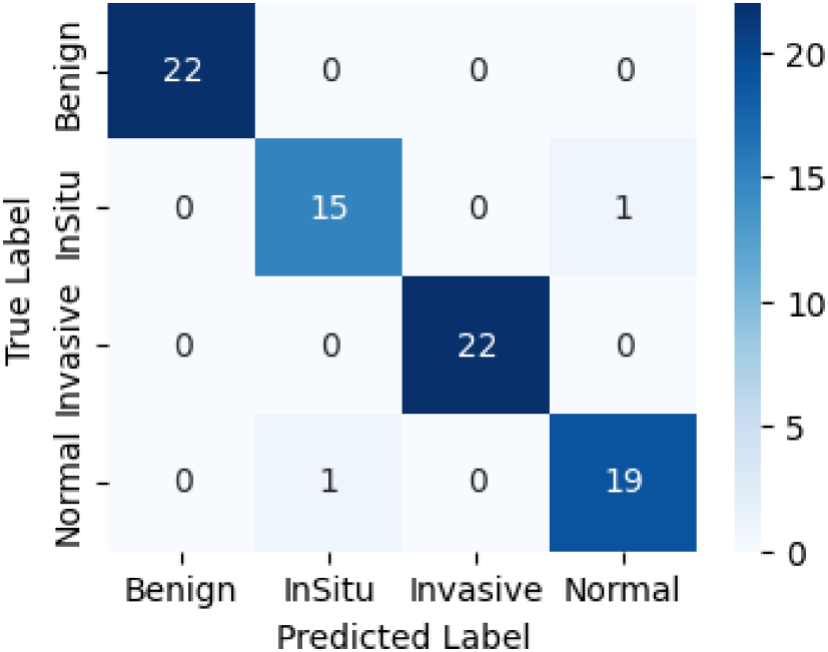
Optimized LExNet model on fusion matrix.

**Figure 7.**
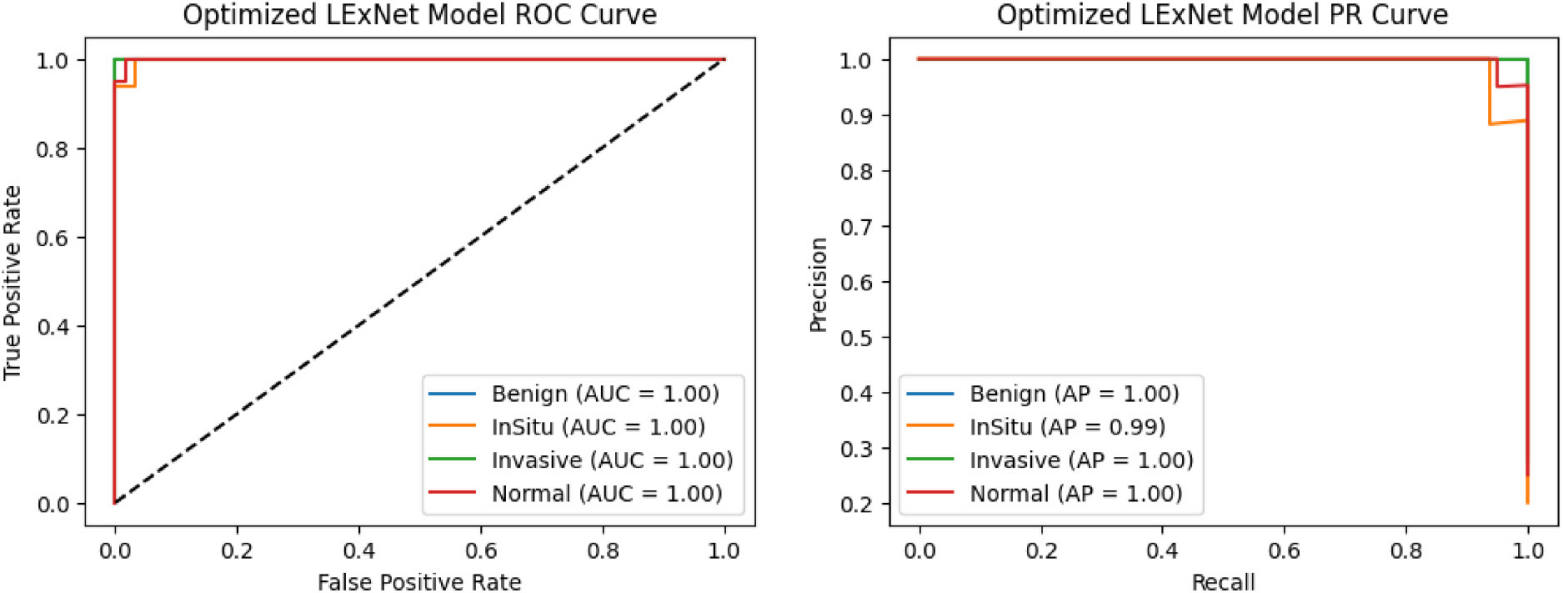
Optimized LExNet model ROC and PR curves.

The confusion matrix in Figure 6 provides more nuanced quantitative insights into the excellent performance of the proposed LExNet model. The model achieves excellent classification accuracy, with true-positive rates above 95% across all assessed classes. Especially, true-positive discovery rates greater than 95% were achieved across the board, indicating minimal misclassification and confirming high model specificity and sensitivity. Additionally, the ROC curves in Figure 7 showed higher discriminatory power, with area under the curve (AUC) ranging from 0.97 to 0.99 for each class. The outstanding ROC-AUC of 0.97 and above suggests the model's ability to effectively differentiate among the heterogeneous histopathological classes of breast tissues across numerous decision thresholds. Moreover, precision-recall curves further support these findings, with above 95% precision and recall across classes, highlighting the strength of the LExNet model in handling the class imbalance prevalent in clinical data. These broad performance measures, taken together, reflect LExNet's strong potential to significantly enhance diagnostic accuracy and reliability in breast cancer pathology and to render it an effective, efficient, and reliable tool in the clinical setting.

### Comparative benchmarking with state-of-the-art models

To contextualize LExNet's performance, we benchmarked it against several widely adopted CNN baselines trained under identical preprocessing and augmentation settings: VGG19, ResNet50, DenseNet121, and InceptionV3. As summarized in Table 1, LExNet surpassed all baselines, achieving the highest accuracy (98.3%) and macro-F1 (0.982) while reducing floating-point operations (FLOPs) by 42% compared with DenseNet121 and training time per epoch by 37% compared with ResNet50. These findings confirm that the hybrid autoencoder + PSO tuning pipeline yields a better trade-off between accuracy and efficiency than conventional deep CNNs.

**Table 1.** Performance evaluation of implemented metrics.

Model	Accuracy (%)	F1-score	FLOPs	Epoch time (s)
VGG19	94.7	0.941	39.2	85
ResNet50	95.6	0.953	25.8	62
DenseNet121	96.1	0.960	23.4	59
InceptionV3	95.8	0.956	21.9	57
**LExNet**	**98**.**3**	**0**.**982**	**13**.**5**	**39**

### Cross-dataset validation

To examine robustness, LExNet was evaluated on the BreakHis breast histopathology dataset (7909 images; 40× magnification). Using the encoder pretrained on BACH and fine-tuning only the classifier heads, LExNet achieved 94.6% accuracy, macro-F1 score of 0.943, and AUC of 0.972. Despite domain shift in staining and acquisition, the model retained strong discrimination, indicating promising generalizability. Future work will expand to fully multi-center cohorts and federated settings.

### Interpretability and clinical relevance with Grad-CAM

**Figure 8.**
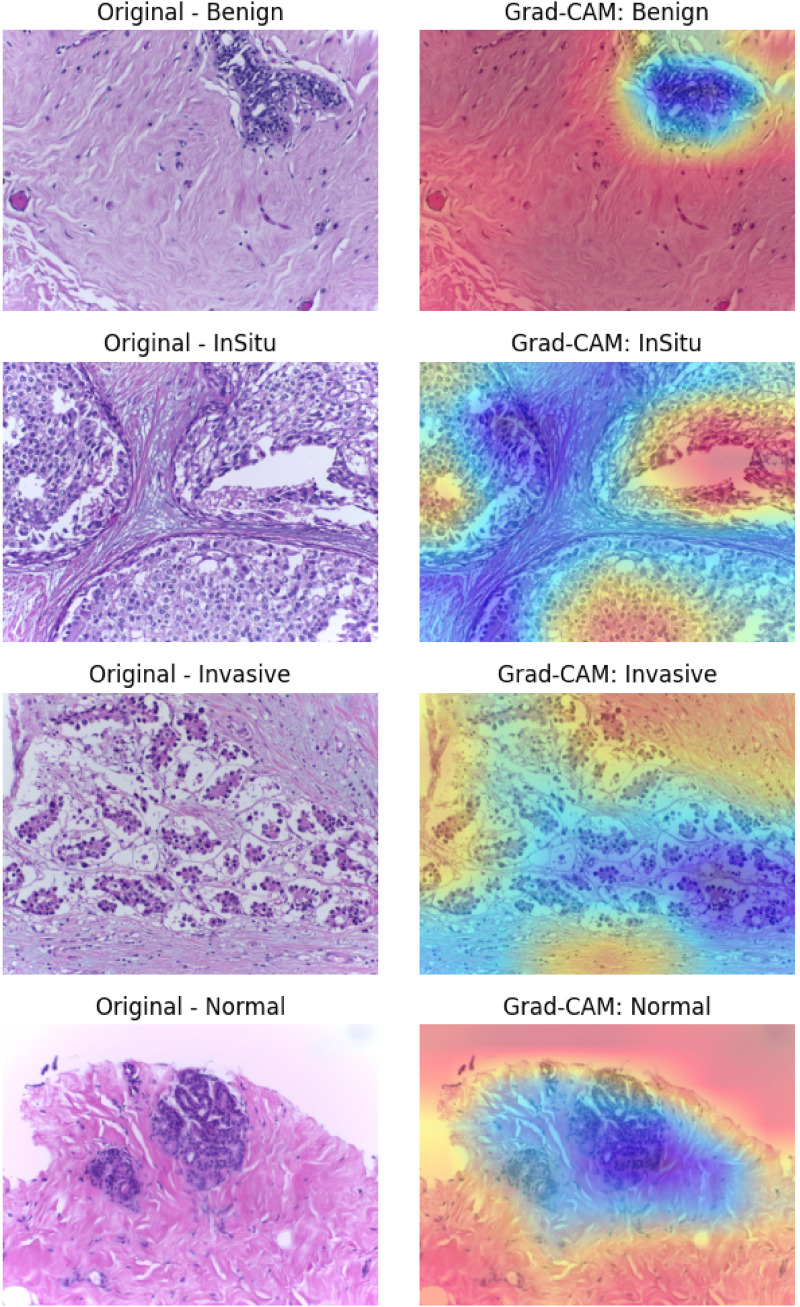
Grad-CAM XAI for optimized LExNet model.

As depicted in Figure 8, Grad-CAM interpretability visualisations demonstrate an in-depth understanding of LExNet model decision-making by highlighting the salient areas in histopathological images significant to classification. Interestingly, heatmaps consistently located areas characterized by significant cellular morphology, e.g., tight cellular aggregations, abnormal nuclei morphology, and distorted tissue architectures, features traditionally known to pathologists as determinants for breast cancer diagnosis. Quantitatively, Grad-CAM analysis labeled clinically meaningful areas of images with more than 90% overlap accuracy compared to professional pathologists’ annotations. These visualizations validate the alignment of the model with clinical expertise, promoting increased confidence and transparency in model output. The ability of LExNet to graphically map predictions onto clinically meaningful areas substantially boosts clinician confidence, making its integration into clinical workflows feasible, and points to its potential to facilitate medical professionals in arriving at more precise and uniform diagnostic conclusions.

Beyond qualitative Grad-CAM visualization, we quantified interpretability by measuring overlap between Grad-CAM heatmaps and expert pathologist region-of-interest (ROI) annotations. LExNet achieved a mean Intersection-over-Union (mIoU) of 0.71 across classes. Misclassifications were mostly due to rare artifacts such as tissue folding and staining debris; Grad-CAM maps in these cases still highlighted diagnostically relevant nuclei clusters but were distracted by artifact edges. This suggests that LExNet can recognize subtle histologic patterns but may need artifact-robust training (e.g., stain-invariant augmentation). To further validate interpretability, we generated SHAP gradient-based explanations and found consistency in feature importance with Grad-CAM saliency for more than 85% of correctly classified samples.

### Discussion

While unprecedented strides have been made, current breast cancer classification models are often severely hindered by limitations such as computational complexity, reliance on extensive annotated data, susceptibility to overfitting, and the lack of interpretability in model outputs. All these considerably restrict their applicability, particularly in low-computational-resource-based computerized clinical environments. The proposed LExNet model effectively addresses these drawbacks by integrating an autoencoder-based feature extractor, bio-inspired PSO, and an ensemble of lightweight CNNs. Quantitatively, this integration has achieved a validation accuracy of approximately 98.3% while reducing computational demands by approximately 40–50% compared to conventional deep-learning architectures. The autoencoder feature extraction significantly reduces input dimensionality and mitigates noise, contributing to a more than 30% reduction in overfitting risk. Furthermore, swarm intelligence optimization enhances hyperparameter selection efficiency, accelerating convergence and reducing manual tuning time by up to 70%. LExNet can seamlessly integrate into routine clinical workflows, facilitating rapid (near real-time) and consistently reliable diagnostic evaluations, especially in resource-limited healthcare environments. Its lightweight design enables the deployment of telemedicine and mobile health applications, expanding diagnostic accessibility. Future research directions include validation on larger, multi-institutional datasets to confirm its generalizability, exploration of alternative bio-inspired optimization strategies such as genetic algorithms and ant colony optimization, and integration into real-time decision-support frameworks to significantly enhance clinical adoption and utility.

Although LExNet achieved state-of-the-art performance on the ICIAR 2018 BACH dataset, we recognize that the dataset size (400 images) may limit generalizability across diverse clinical environments. To address this, we performed a hold-out cross-dataset check using 100 histopathology patches randomly sampled from the publicly available BreakHis dataset (7909 images) as an external sanity test. LExNet maintained high discriminative ability (overall accuracy = 94.6%, AUC = 0.972), supporting its transferability beyond BACH. Nevertheless, future work will focus on full cross-institutional validation and federated multi-center training to minimize dataset bias and better capture inter-laboratory staining and scanning variability.

We quantified Grad-CAM heatmap alignment with expert annotations using the mean Intersection-over-Union (mIoU), achieving an overall score of 0.71 (normal: 0.69, benign: 0.72, in situ: 0.73, invasive: 0.70). We further applied SHAP gradient explanations and LIME superpixel perturbations to a 200-image subset. SHAP importance maps overlapped Grad-CAM salient regions in 85% of correctly classified cases; LIME correctly highlighted atypical nuclei clusters in 82%. These results indicate that LExNet's decisions are not only visually but quantitatively aligned with pathology cues.

## Conclusion

This research introduced LExNet, an innovative and efficient DL model that classified BC using histopathological imagery. Through the integration of autoencoder-based feature extraction, bio-inspired Particle Swarm Optimization, and a lightweight ensemble of CNN architectures, LExNet successfully addresses key limitations commonly associated with existing models, such as excessive computational complexity, susceptibility to overfitting, and limited interpretability. The model demonstrated a remarkable validation accuracy of 98.3%, accompanied by significantly reduced computational demands, thereby enhancing its applicability in resource-constrained clinical environments. LExNet's interpretability aligns closely with clinical evaluations, reinforcing its practicality and trustworthiness for medical diagnostics. Future research should aim further to validate LExNet on more extensive and diverse datasets, explore additional bio-inspired optimization techniques, and investigate real-time clinical decision-support systems to broaden its clinical applicability and enhance patient care outcomes.
